# Neutron Diffraction Evaluation of Near Surface Residual Stresses at Welds in 1300 MPa Yield Strength Steel

**DOI:** 10.3390/ma10060593

**Published:** 2017-05-29

**Authors:** Ebrahim Harati, Leif Karlsson, Lars-Erik Svensson, Thilo Pirling, Kamellia Dalaei

**Affiliations:** 1 Department of Engineering Science, University West, SE-461 86 Trollhättan, Sweden; leif.karlsson@hv.se (L.K.); lars-erik.svensson@hv.se (L.-E.S.); 2 Institut Max von Laue-Paul Langevin, 6 rue Jules Horowitz, BP156, F-38042 Grenoble, France; pirling@ill.fr; 3 ESAB AB, Lindholmsallen 9, 40227 Gothenburg, Sweden; kamellia.dalaei@esab.se

**Keywords:** residual stress, high strength steel, neutron diffraction, weld toe, low transformation temperature welding consumable

## Abstract

Evaluation of residual stress in the weld toe region is of critical importance. In this paper, the residual stress distribution both near the surface and in depth around the weld toe was investigated using neutron diffraction, complemented with X-ray diffraction. Measurements were done on a 1300 MPa yield strength steel welded using a Low Transformation Temperature (LTT) consumable. Near surface residual stresses, as close as 39 µm below the surface, were measured using neutron diffraction and evaluated by applying a near surface data correction technique. Very steep surface stress gradients within 0.5 mm of the surface were found both at the weld toe and 2 mm into the heat affected zone (HAZ). Neutron results showed that the LTT consumable was capable of inducing near surface compressive residual stresses in all directions at the weld toe. It is concluded that there are very steep stress gradients both transverse to the weld toe line and in the depth direction, at the weld toe in LTT welds. Residual stress in the base material a few millimeters from the weld toe can be very different from the stress at the weld toe. Care must, therefore, be exercised when relating the residual stress to fatigue strength in LTT welds.

## 1. Introduction

The weld toe is the most probable fatigue crack initiation location in welded parts. This is mainly due to the local stress concentration caused by geometrical changes and tensile residual stresses induced by welding. Presence of crack-like imperfections and cold laps in the weld toe will also make the weld toe a critical location for cracking. Therefore, determination of residual stresses in the weld toe region is of particular importance [1,2]. 

X-ray diffraction is one of the most common methods for measuring residual stresses at welds but, due to the low penetration depth of X-rays, such measurements only give information about the stresses near the surface (typically tens of micrometres [3]). Other techniques, such as neutron diffraction, are therefore necessary to measure the residual stress, both near the surface and in the interior of components (up to several cm) [4,5,6,7]. A number of studies have been carried out to measure residual stresses of welds by neutron diffraction [8,9,10,11,12,13,14,15]. However, most of the previous studies have measured residual stresses either in the base material at some distance from the weld toe or at some depth below the surface at the weld toe [8,9,16,17]. Only one study has reported near surface residual stresses at the weld toe [18]. Ramjaun et al. [18] measured residual stresses using neutron diffraction at 0.15 mm (averaged over 0–0.3 mm below surface) and 2.5 mm below the surface in a welded 700 MPa yield strength steel. They observed very steep residual stress gradients across the weld toe 0.15 mm below the top surface.

Application of Low Transformation Temperature (LTT) consumables in welding is a recent approach to increase the fatigue strength of welds. The increase in fatigue strength gained using these consumables is due to the volume expansion caused by the transformation of austenite to martensite at low temperatures, typically around 200 °C. This lowers the tensile residual stresses and may even produce compressive residual stresses within the weld region, particularly at the weld toe [19,20,21,22,23]. In previous studies [1,24,25] fatigue properties of welded parts produced using LTT or conventional consumables in steels with yield strengths ranging from 650 to 1300 MPa have been investigated. LTT welds were always found to achieve higher fatigue strengths than conventional welds for steels with yield strengths up to 1021 MPa. This higher fatigue strength of LTT welds were related to lower residual stresses. However, these were measured 1 mm away from the weld toe, using X-ray diffraction [1]. For 1300 MPa steels and for the fatigue testing procedure used, a bit surprisingly no difference between LTT and conventional welds in fatigue properties were found [26]. Thus, it is assumed that a more detailed evaluation of residual stresses locally at the weld toe would contribute to a better understanding of the fatigue behaviour of LTT and conventional welds.

The main aim of this paper is to evaluate the residual stress distribution both near the surface and in depth around the weld toe in a 1300 MPa yield strength steel welded using an LTT consumable. The LTT weld, produced in a high strength steel, was selected as previous studies [18] suggest there might be a significant stress gradient locally at the weld toe. Neutron diffraction was the main measurement method, which was complemented with X-ray diffraction. Residual stresses at different positions are compared and used to explain the LTT effect on fatigue properties. The relevance of residual stress measurement location in prediction of fatigue strength is also discussed.

## 2. Materials and Methods

### 2.1. Base and Filler Material

Weldox 1300 plates with a thickness of 15 mm was used as base material. An experimentally designed metal cored wire with high amounts of Cr and Ni to produce a low martensite start (M_s_) temperature was used as LTT filler material. The chemical compositions of the base and filler materials are given in Table 1. Yield and tensile strengths of the base material and all-weld metal are summarized in Table 2.

### 2.2. Welding

**Figure 1:**
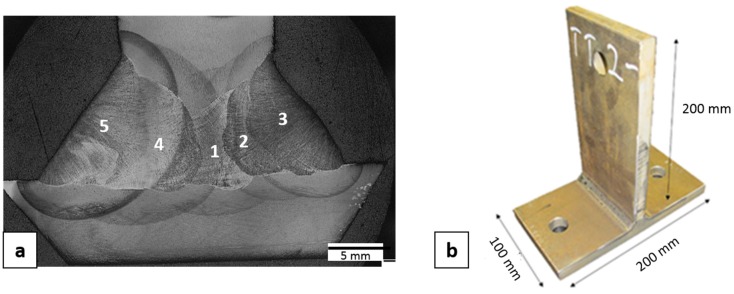
(**a**) The welding sequence with five beads; (**b**) Design and dimension of T-shaped sample used in residual stress measurements.

Welded assemblies were produced by joining two plates with dimensions of 500 × 200 × 15 mm^3^. Robotic Gas Metal Arc Welding (GMAW) with Ar + 18% CO_2_ as shielding gas, was used to produce full penetration fillet welds from both sides in five beads as shown in Figure 1a. The welded assemblies were sliced and machined to produce specimens, with dimensions as shown in Figure 1b. The pre-heat temperature used was 75 °C and the maximum inter-pass temperature was 125 °C. The welding parameters are summarized in Table 3.

### 2.3. Neutron Diffraction Stressmeasurements

**Figure 2:**
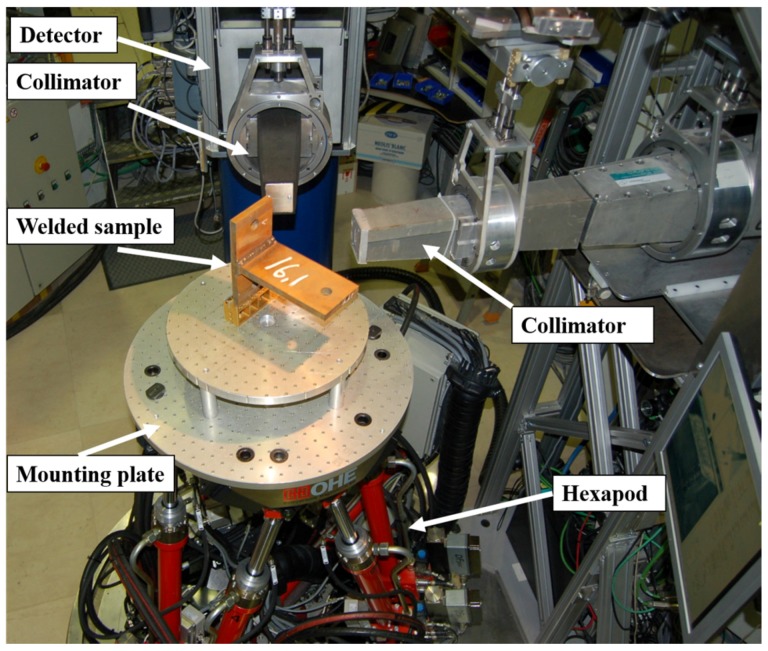
Experimental setup for residual stress measurements in a T-shaped welded sample in the SALSA instrument.

Neutron diffraction measurements were performed with the SALSA (Strain Analyser for Large and Small scale engineering Applications) instrument at Institut Laue-Langevin (ILL) located in Grenoble, France. SALSA is a monochromatic strain diffractometer dedicated to measure strains. The experimental setup used for stress measurements in a T-shaped welded sample is shown in Figure 2. As shown in the figure, SALSA uses a hexapod as sample manipulator. This is basically a robot with six degrees of freedom achieved by using six independent hydraulically controlled pistons. The load capacity of the SALSA hexapod is in excess of 500 kg and it allows tilts up to 30° and a translation range of 600 mm. The positioning precision will vary between 20 and 50 µm, depending on the load and position in space [27,28].

#### 2.3.1. Measurement of Stress-Free Lattice Parameter 

**Figure 3:**
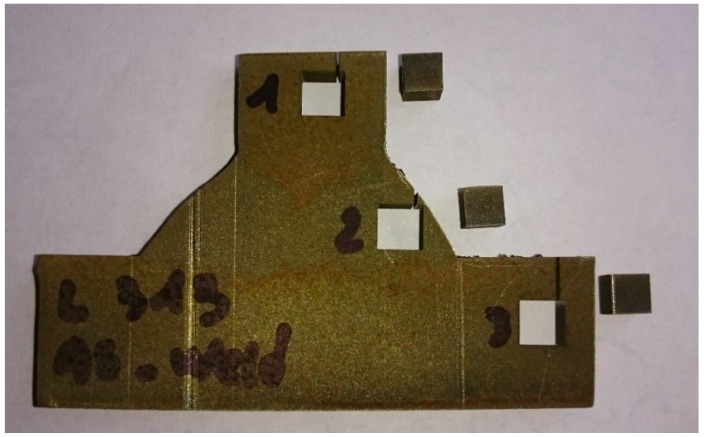
Cubes machined from the base and weld metal (stress-free sample) used for the reference lattice spacing (*d*_0_) measurements.

Measurement of lattice spacing (*d*_0_) in the stress-free condition was needed for calculation of strains. To achieve this, a plate was cut from an identical welded sample and then three cubes from base and weld metals with 4 × 4 × 4 mm^3^ were machined by electro-erosion, (Figure 3). The cube machined from the weld region was used for residual stress evaluation in the weld (not reported in this paper). 

#### 2.3.2. Stress Measurements and Calculations

**Figure 4:**
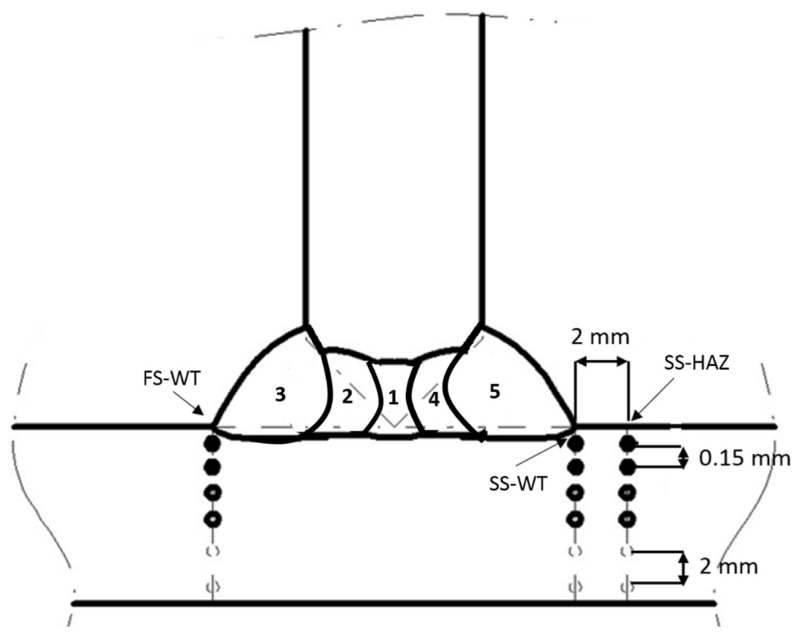
Location of measurement positions. Distance between two points are 0.15 mm close to the surface and gradually increases to 2 mm at greater depth.

**Figure 5:**
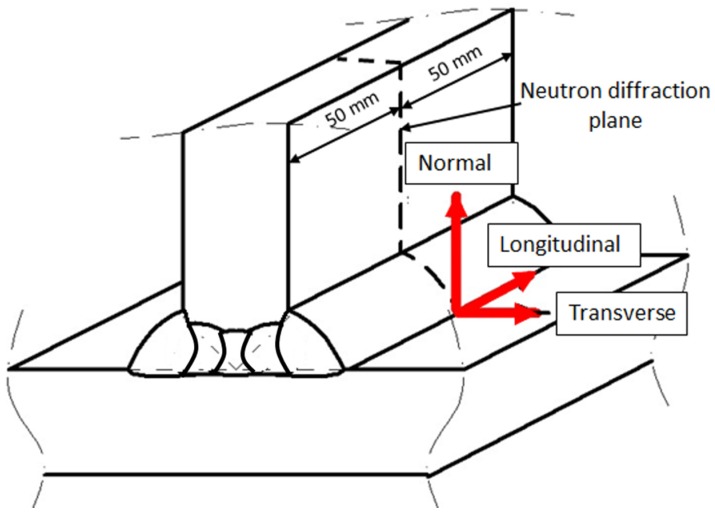
Welded specimen showing the three directions of stress measurements and the neutron diffraction plane.

Residual stress measurements were done from surface to depth in the base material for positions FS-WT (first welded side at the weld toe), SS-WT (second welded side at the weld toe) and SS-HAZ (second welded side, 2 mm away from the weld toe into HAZ) as shown in Figure 4. To achieve this, neutron scanning was performed by moving the gauge volume from surface to points inside the material in steps of 0.15 mm and in greater steps inside the material as shown in the figure. Longitudinal, transverse, and normal stresses were measured based on the assumption that these directions, by symmetry, are the principal stress directions as shown in Figure 5.

The inter-planar spacing (*d*) of the Fe {211} crystallographic plane at the detector angle, 2*θ*, of approximately 101° was chosen for measurements of all the principal stress directions. The gauge volume dimension was determined by using slits in front of the incoming beam and collimating the diffracted beam. A gauge volume of 0.6 × 0.6 × 10 mm^3^ was used for the normal and transverse stress measurement, while a gauge volume of 0.6 × 0.6 × 2 mm^3^ mm was used in the longitudinal direction. The increase in gauge volume along the welding direction, in the normal and transverse directions, allows faster measurement and at the same time having high intensity of neutrons. The used wavelength was 1.8 Ångström.

The lattice spacing *d* is related to the scattering angle *θ* by Bragg’s law as shown in Equation (1).

(1) $$\lambda =2d\sin\left(\frac{\theta }{2}\right)$$

where *λ* is the wavelength. Gaussian fitting was used to fit the intensity profile and precise determination of the peak position. Stress-free lattice spacing (*d*_0_) measurements combined with the lattice spacing measurements were used to calculate strain (*ε*) using Equation (2).

(2) $$\varepsilon_{hkl}=\left(\frac{d_{hkl}}{d_{0hkl}}-1\right)=-\cot\theta \times \Delta \theta$$

where *ε* is strain, *hkl* are the coordinate planes, *d* is the lattice spacing, *d*_0_ is strain-free spacing and Δθ is peak shift. The principal stress ($\sigma_{ii}$) could be analysed once the strain was determined, using the Hooke’s law for three dimensional state of stress as shown in Equation (3).

(3) $$\sigma_{ii}=\frac{E}{\left(1+\nu \right)}\left[\varepsilon_{ii}+\frac{\nu }{\left(1-2\nu \right)}\left(\varepsilon_{11}+\varepsilon_{22}+\varepsilon_{33}\right)\right]$$

where Ε and υ are the Young’s modulus and Poisson’s ratio, respectively, and *i* = 1, 2, 3 indicates the component of stress and strain relative to the principal stress directions. Elastic constants values of Ε = 210 GPa and υ = 0.33 were used to calculate stress from measured strains.

#### 2.3.3. Near Surface Stress Correction

Near the surface the gauge volume is only partially penetrating into the material. The surface position with respect to the gauge volume was determined with the known intensity profile. When the gauge volume near a surface is only partially penetrating into the material, the effective position of strain information is geometrically displaced from the centre of the gauge volume. In order to correct these effects a computer program was developed using Mathcad based on intensity-weighed centre of gravity calculations. This enabled the determination of the centre of the diffracting volume inside the gauge volume for each step of the scan, taking into account the size and shape of the gauge volume, the exact position of the sample relative to the neutron beam, and different paths of the neutrons within the sample [29,30].

### 2.4. Residual Stress Measurements Using X-ray Diffraction

Residual stresses were measured for an identical LTT sample in the transverse and longitudinal directions relative to the weld toe. The measurements were done using X-ray diffraction with Stresstech X3000 X-ray equipment (StressTech, Vaajakosk, Finland). The ${sin}^{2}\varphi$ method was used with Cr-Kα radiation for {211} planes in the ferrite phase and the φ angle was varied between −40° and +40° with ±5° oscillations (more than 15 angles in total). Measurements were performed at distances of 0.5, 2 and 4 mm from the weld toe in the base material. A collimator with a size of 3 mm × 1 mm was used such that the 3 mm side was oriented parallel to the weld toe fusion boundary.

## 3. Results

### 3.1. Neutron Diffraction

**Figure 6:**
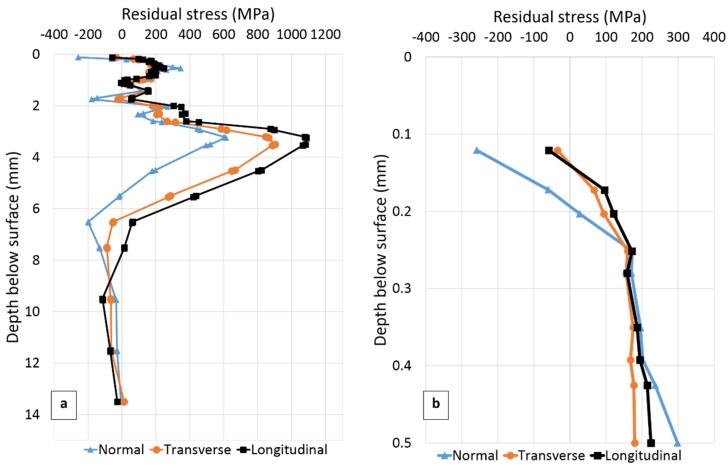
(**a**) Residual stresses as function of distance below the plate surface at the weld toe on the second welded side (SS-WT in Figure 4); (**b**) Residual stress from surface to 0.5 mm below the surface. Note the compressive residual stresses near the surface.

**Figure 7:**
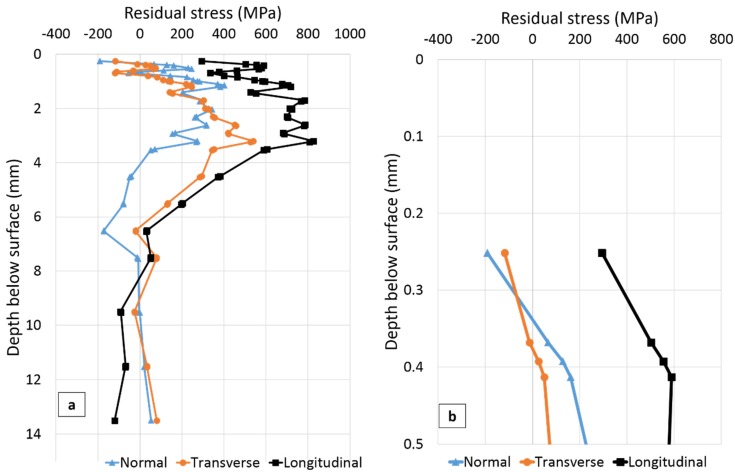
(**a**) Residual stresses as function of distance below the plate surface on the second welded side 2 mm away from the weld toe (SS-HAZ in Figure 4); (**b**) Residual stress from surface to 0.5 mm below the surface.

**Figure 8:**
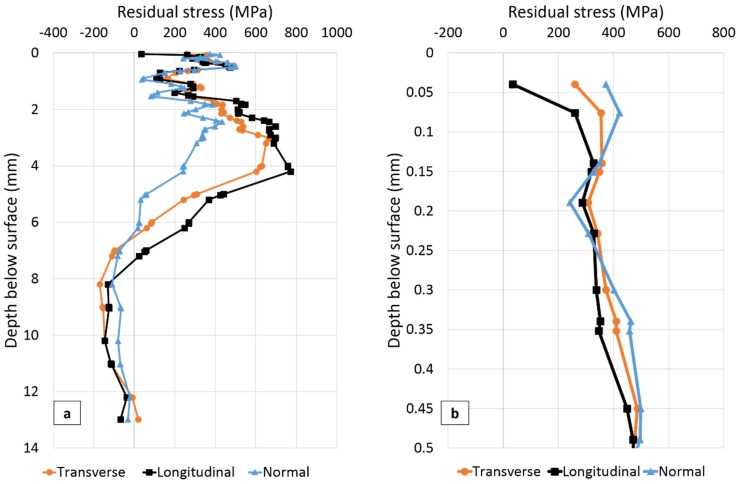
(**a**) Residual stresses as function of distance below the plate surface at the weld toe on the first welded side (FS-WT in Figure 4); (**b**) Residual stress from surface to 0.5 mm below the surface.

The residual stresses measured in three directions at the three positions SS-WT, SS-HAZ and FS-WT (Figure 4) as a function of distance from the plate surface are presented in Figure 6, Figure 7 and Figure 8, respectively. The residual stress profile from surface to 0.5 mm below the top surface is magnified for each figure and presented in Figure 6b, Figure 7b and Figure 8b.

In Figure 6, the point closest to the surface for which residual stress was measured is 120 µm below the surface. The experimental uncertainties vary from about ±140 MPa close to the weld toe surface to ±30 MPa at greater depths. 

A summary of observations extracted from Figure 6 is given in Table 4.

From Figure 6 and Table 4, it can be seen that compressive residual stresses 120 µm below the surface are present in all directions being highest in the normal direction. From the figure, it can also be seen that in general almost the same residual stress distribution is seen in the transverse, longitudinal and normal orientations. A relatively large change with depth in residual stress can be observed within 7 mm below the surface, in all directions. Maximum tensile stresses of about 900 MPa 3.5 mm below the surface, in the transverse, about 1090 MPa 3.2 mm below the surface, in the longitudinal and about 610 MPa 3.2 mm below the surface in the normal direction can be seen. Stress then decreases and is fairly constant from 5 to 8 mm and approaches zero at 12 mm from the surface.

Another interesting observation from Figure 6 and Table 4 can be made regarding the near surface stress gradients. Steep stress gradients very close to the surface are evident especially in the normal direction. 

In Figure 7, the closest point to the surface for which residual stress was measured is 250 µm below the surface. The experimental uncertainties vary from about ±100 MPa close to the weld toe surface to ±30 MPa at greater depths. Interesting observations extracted from Figure 7 are summarized in Table 5.

From Figure 7 and Table 5, the presence of steep near surface stress gradients is evident. It can also be seen that in the normal and transverse orientations the stress 250 µm below the surface is compressive while in the longitudinal direction it is tensile. In the longitudinal direction stress remains tensile, with a maximum of about 830 MPa 3.2 mm below the surface, until it changes to compressive below about 9.5 mm depth from the surface.

A relatively large variation in residual stress can be observed very close to the surface to about 7 mm depth below the surface, in all directions (Figure 7). 

In Figure 8, the closest point to the surface for which residual stress was measured is 39 µm below the surface. The experimental uncertainties vary from about ±120 MPa close to the weld toe surface to ±30 MPa at greater depths. Interesting observations extracted from the information in Figure 8 is shown in Table 6.

From Figure 8 and Table 6 it can be seen that residual stress is tensile in all three orientations 39 µm below the surface. Almost the same near surface stress distribution are observed in all directions (Figure 8b). Similar to stress distributions in positions SS-WT and SS-HAZ, steep near surface stress gradients and large variation of stress within 7 mm below the top surface are observed. 

### 3.2. X-ray Diffraction

**Figure 9:**
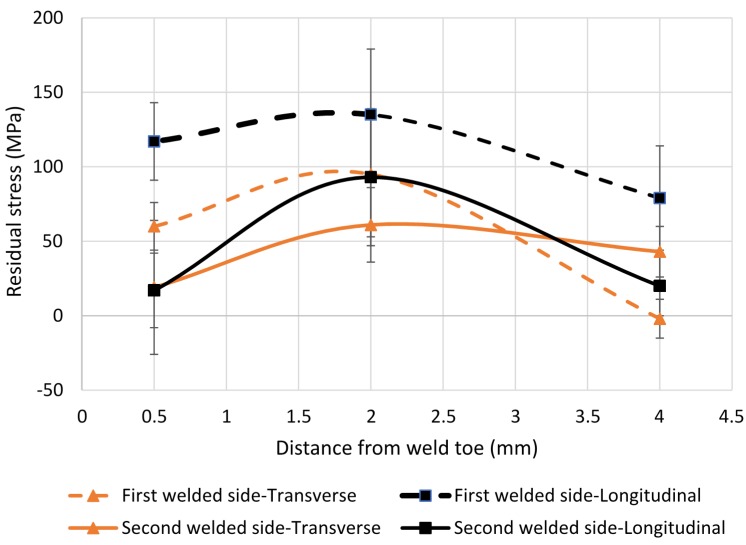
Transverse and longitudinal surface residual stresses close to the weld toe.

Surface residual stress as a function of distance from the weld toe in the LTT sample on the first and second welded sides are shown in Figure 9. 

It can be seen from Figure 9 that the stress is tensile in transverse and longitudinal orientations on both the first and second welded sides at all distances from the weld toe (except the transverse stress in the first welded side 4 mm from the weld toe where the stress is −4 MPa). Lower tensile stresses are observed on the second welded side compared to those on the first welded side 0.5 mm away from the weld toe. On the first welded side, lower transverse residual stresses of about 60 MPa is observed compared to about 120 MPa in the longitudinal direction. However, on the second welded side almost the same values for residual stress (about 20 MPa) in transverse and longitudinal directions can be seen 0.5 mm from the weld toe. 

## 4. Discussion

The main aim of this paper is to evaluate the residual stress distribution near the surface and as a function of depth around the weld toe in a 1300 MPa yield strength steel welded using an LTT consumable. Neutron diffraction was used as the main measurement method and X-ray diffraction as a complementary method. Residual stresses at different positions are compared and used to explain the LTT effect on fatigue properties. The relevance of residual stress measurement location in prediction of fatigue strength will also be discussed. 

### 4.1. Stress Distribution

A summary of the stress at the measurement point closest to the surface, near surface residual stress gradient and maximum tensile stress for the three positions (see Figure 4) in the transverse (T), longitudinal (L) and normal (N) directions, is given in Table 7 for further discussion.

From Table 7 it can be seen that very steep surface stress gradients were observed in all positions and directions with the largest gradients occurring between the two measuring points closest to the surface. These findings can be discussed in the light of the results obtained by Ramjaun et al. [18] where they measured residual stresses 0.15 mm below the surface at the weld toe region in butt welds produced with an LTT consumable, using neutron diffraction. Steep and localized residual stress gradients of about 400 MPa/mm in the transverse, 500 MPa/mm in the normal and 1000 MPa/mm in the longitudinal directions were observed across the weld toe. Interestingly, enough these are of the same magnitude as the transverse and longitudinal stress gradients found in this study but in the depth direction for position SS-WT. In a study by Camilleri et al. [31] a relatively sharp change in residual stresses was predicted around the fusion boundary at mid-thickness in LTT welds. Using simulation, longitudinal residual stresses as a function of distance from the fusion boundary were calculated for LTT and conventional welds. Results of their work showed a relatively local sharp change in residual stresses from 350 MPa to −50 MPa in a few millimetres from the fusion boundary for the LTT weld whereas no significant change in residual stresses were predicted for the conventional welds. Thus, it can be concluded that there is a very local stress effect with large gradients both transverse to the weld toe line and in the depth direction, at the weld toe in LTT welds.

In the study by Ramjaun et al. [18] residual stress was also measured 2.5 mm below the surface. Stress gradients of about 120 MPa/mm in the transverse, 80 MPa/mm in the longitudinal and 40 MPa/mm in the normal directions can be calculated from the stress results 0.15 mm and 2.5 mm below the surface. Almost the same stress gradient of 120 MPa/mm were also observed in the present study in the transverse direction, calculated from the stress 0.12 mm and 2.5 mm below the surface. However, much steeper stress gradients in the longitudinal (175 MPa/mm) and in the normal (177 MPa/mm) directions were found. The different weld geometries may explain the different stress gradients as suggested by results from Takahashi and Yasuda [9]. They measured very different residual stresses 3 mm below the weld toe of butt and cruciform welds both produced using the same LTT filler material. Neutron diffraction showed residual stresses of about 400 MPa in the transverse, 700 MPa in the longitudinal and zero in the normal direction for the butt welds while about −200 MPa in the transverse, −300 MPa in the longitudinal and zero in the normal direction were observed for cruciform welds. 

Compressive residual stresses are present in the second welded side while tensile stresses are found in the first welded side (Figure 6 and Figure 8, Table 7). X-ray results (Figure 9) also present lower tensile residual stresses in the second welded side 0.5 mm from the weld toe. This is most probably related to the tempering effect on the first welded side when welding the second side. These results supports the findings that fatigue crack initiation and propagation preferentially occurred at the first welded side in previous work studying similar welded samples [32]. 

### 4.2. X-ray Diffraction

**Figure 10:**
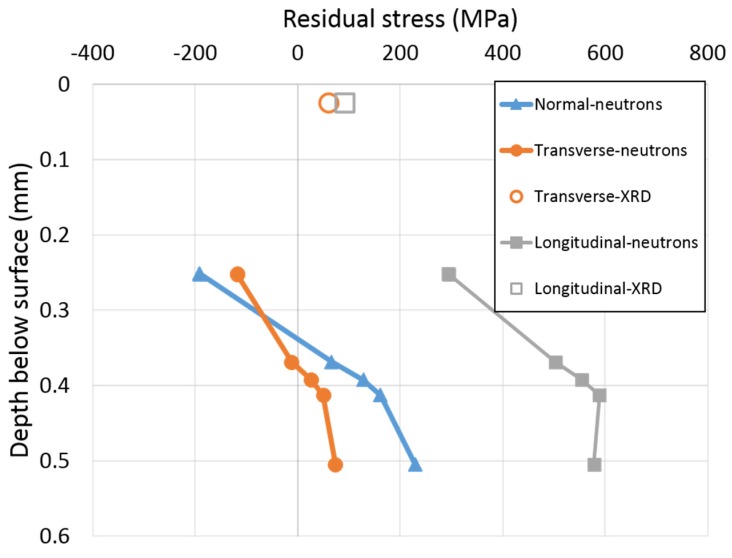
Near surface residual stress obtained 2 mm away from the weld toe (SS-HAZ) using X-ray and neutron diffraction.

Near surface residual stress results obtained 2 mm away from the weld toe (SS-HAZ) using X-ray and neutron diffraction are summarized in Figure 10. As can be seen from the figure, different residual stresses were measured using the two methods. The different results obtained by X-ray and neutron diffraction methods can be related to the fact that the two techniques did not extract data from the same depth. Since no data are available at depths between the X-ray and neutron results only assumptions can be made about the residual stress distribution within 250 µm below the surface. 

However, although speculative, the stress trend in position SS-WT (see Figure 6b) suggests that the stress gradient in position SS-HAZ would most probably result in a stress minimum 120 µm or closer below the surface. From Figure 10, about 200 MPa difference in residual stress can be seen comparing the stresses obtained using X-ray and neutron diffraction in both transverse and longitudinal directions. These results can be compared with the findings reported by Ramjaun et al. [18] where they measured surface residual stresses using X-ray and neutron diffraction 0.15 mm below the surface. Very similar residual stresses were observed by the two methods in the transverse direction but a quite large difference (200 MPa) was seen in the longitudinal direction, at some distance from the weld toe.

Comparing residual stresses measured using X-ray and neutron diffraction, it is, therefore, evident that the distribution of near surface residual stress is very complicated. Nevertheless, steep stress gradients are expected within 250 µm below the surface in all three directions. 

### 4.3. LTT Effect

The effectiveness of an LTT consumable in increasing the fatigue strength is due to its ability to reduce tensile residual stresses or even produce compressive residual stresses in the weld toe region. Near surface (120 µm below the top surface) residual stresses obtained in this study (Figure 6) suggests that the LTT consumable has produced compressive residual stresses in all directions, very close to the surface at the weld toe. However, lower compressive stresses were found in this study compared to the results reported by Ramjaun et al. [18], though the used base materials and weld geometries are different. They measured compressive residual stresses of about −600 MPa in transverse, −400 MPa in longitudinal and −300 MPa in the normal directions. These are much higher than the corresponding compressive stresses measured in this study of −35 MPa, −58 MPa and −258 MPa. The effect of transformation start and finish temperatures of the base and weld metals on the final residual stress state was discussed in [32]. It was suggested that when the LTT weld starts to transform to martensite the surrounding base metal is only partly transformed to martensite for a 1300 MPa yield strength steel while transformation to martensite is more or less completed when the base material is a lower strength steel. This might be a reason for the lower compressive residual stresses in the weld toe compared to those in the referred study [18] where an 800 MPa yield strength steel was used. The effectiveness of LTT consumables in reducing residual stresses is obviously depending on a number of factors such as steel strength (composition) as suggested here, geometry as discussed above and tempering from welding of adjacent weld beads as suggested by comparison of the first and second welded sides.

### 4.4. Relevance of Residual Stress Measurement Location in Prediction of Fatigue Strength

The present paper shows that there is a steep residual stress gradient both transverse to the weld toe line and in the depth direction, at the weld toe in LTT welds. Neutron diffraction complemented by X-ray diffraction provided a better understanding of the presence of the complicated and large near surface stress gradients around the weld toe in the depth direction. In an earlier paper [1] the relative effects of residual stresses, measured by X-ray diffraction, and weld toe radius on fatigue strength of welds produced using LTT or conventional filler materials in single- and two-pass welds were examined. As expected, fatigue strength of LTT welds was higher than that of conventional welds and two-pass LTT welds showed higher fatigue strength than single-pass LTT welds. Having almost the same measured weld toe radius for the single- and two-pass LTT welds, the higher fatigue strength of two-pass LTT welds was concluded to be related to a lower residual stress at the weld toe. However, larger tensile residual stresses, 1 mm away from the weld toe, were measured for the two-pass LTT welds. Thus, the residual stresses at the weld toe, which are critical from the fatigue point of view, are most likely very different from the stress 1 mm away.

Due to difficulties and limitations to measure residual stresses locally at the weld toe, especially with X-ray diffraction, most often in the literature and recommendations [33] residual stresses a few millimetre away from the weld toe have been measured and related to fatigue properties. However, as it was stated earlier, different near surface stresses and gradients were observed for positions SS-WT and SS-HAZ. Therefore, care must be exercised when relating the residual stress to fatigue strength, in particular for LTT welds, as stress in the base material a few millimetre from the weld toe can be very different from the stress locally at the weld toe. 

## 5. Conclusions

This paper investigated the residual stress distribution near the surface and as a function of depth around the weld toe in a 1300 MPa yield strength steel welded using an LTT consumable. Neutron diffraction was used as the main measurement method and complementary studies were performed using X-ray diffraction. Based on the results and discussion the following can be concluded:

- Near surface residual stresses, as close as 39 µm below the surface, were measured using neutron diffraction and evaluated by applying a near surface data correction technique based on intensity-weighed centre of gravity calculations.
- Very steep near surface stress gradients within 0.5 mm of the surface were found both at the weld toe and 2 mm into the HAZ.
- Neutron diffraction results showed that the LTT consumable was capable of inducing compressive residual stresses 120 µm below the surface in all directions at the weld toe on the second welded side. Tensile residual stresses were observed 39 µm below the surface at the weld toe of the first welded side.
- The largest tensile residual stresses, as high as 0.84 of the steel yield strength, were seen 2-3 mm below the surface both at the weld toe and 2 mm into the HAZ, being largest in the longitudinal direction.
- Care must be exercised when relating the residual stress to fatigue strength for LTT welds as stress in the base material a few millimetre from the weld toe can be very different from the stress at the weld toe.

## Figures and Tables

**Table 1:** Chemical compositions of base and filler materials (wt. %).

Material		C	Si	Mn	Cr	Ni	Mo
Base material	Weldox 1300	0.25	0.5	1.4	0.8	1.3	0.7
Filler material	LTT ^1^	0.01	0.7	1.50	13.0	6.0	0.01

^1^ nominal all-weld metal composition.

**Table 2:** Yield and tensile strengths of base material and all-weld metal.

Material	f_y_ (MPa)	f_u_ (MPa)
Weldox 1300	1295	1562
LTT	736	1127

**Table 3:** Welding parameters.

Bead No.	Current (A)	Voltage (V)	Welding Speed (mm/s)	Energy Input (kJ/mm)
1	245	27	4	1.6
2, 3, 4 and 5	245	28.5	3.3	2.1

**Table 4:** A summary of the main residual stress results for position SS-WT in the transverse (T), longitudinal (L) and normal (N) directions, extracted from Figure 6.

	T	L	N
Stress 120 µm below the surface (MPa)	−35	−58	−259
Stress gradient within 0.12–0.5 mm below the surface (MPa/mm)	565	747	1469
Max. tensile stress (MPa)	902	1088	612

**Table 5:** A summary of the main residual stress results for position SS-HAZ in the transverse (T), longitudinal (L) and normal (N) directions, extracted from Figure 7.

	T	L	N
Stress 250 µm below the surface (MPa)	−118	294	−192
Stress gradient within 0.25–0.5 mm below the surface (MPa/mm)	829	1123	835
Max. tensile stress (MPa)	540	827	401

**Table 6:** A summary of the main residual stress results for position FS-WT in the transverse (T), longitudinal (L) and normal (N) directions, extracted from Figure 8.

	T	L	N
Stress 39 µm below the surface (MPa)	260	34	372
Stress gradient within 0.039–0.5 mm below the surface (MPa/mm)	429	929	216
Max. tensile stress (MPa)	669	771	434

**Table 7:** A summary of the stress at the point closest to the surface, near surface residual stress gradient and maximum tensile stress for the three positions in the transverse (T), longitudinal (L) and normal (N) directions.

	T			L			N		
	FS-WT	SS-WT	SS-HAZ	FS-WT	SS-WT	SS-HAZ	FS-WT	SS-WT	SS-HAZ
Stress at point closest to the surface (MPa)	260	−35	−118	34	−58	294	372	−259	−192
Stress gradient from point closest to the surface to 0.5 mm below the surface (MPa/mm)	429	565	829	929	747	1123	216	1469	835
Stress gradient between the two points closest to the surface (MPa/mm)	2615	1978	904	6253	3015	1785	1396	3881	2024
Max. tensile stress (MPa)/at depth (mm)	669/3.0	902/3.5	540/3.2	771/4.2	1088/3.2	827/3.2	434/2.4	612/3.2	401/1.1
